# Magnetic Resonance Imaging Features under Deep Learning Algorithms in Evaluated Cerebral Protection of Craniotomy Evacuation of Hematoma under Propofol Anesthesia

**DOI:** 10.1155/2021/2209527

**Published:** 2021-10-01

**Authors:** Deqian Xin, Zhongzhe An, Juan Ding, Zhi Li, Leyan Qiao

**Affiliations:** ^1^Department of Anesthesiology, Yantai Yuhuangding Hospital, Yantai 264000, Shandong, China; ^2^Department of Allergy, Yantai Yuhuangding Hospital, Yantai 264000, Shandong, China

## Abstract

This study aimed to explore the value of magnetic resonance imaging (MRI) features based on deep learning super-resolution algorithms in evaluating the value of propofol anesthesia for brain protection of patients undergoing craniotomy evacuation of the hematoma. An optimized super-resolution algorithm was obtained through the multiscale network reconstruction model based on the traditional algorithm. A total of 100 patients undergoing craniotomy evacuation of hematoma were recruited and rolled into sevoflurane control group and propofol experimental group. Both were evaluated using diffusion tensor imaging (DTI) images based on deep learning super-resolution algorithms. The results showed that the fractional anisotropic image (FA) value of the hind limb corticospinal tract of the affected side of the internal capsule of the experimental group after the operation was 0.67 ± 0.28. The National Institute of Health Stroke Scale (NIHSS) score was 6.14 ± 3.29. The oxygen saturation in jugular venous (SjvO_2_) at T4 and T5 was 61.93 ± 6.58% and 59.38 ± 6.2%, respectively, and cerebral oxygen uptake rate (CO_2_ER) was 31.12 ± 6.07% and 35.83 ± 7.91%, respectively. The difference in jugular venous oxygen (Da-jvO_2_) at T3, T4, and T5 was 63.28 ± 10.15 mL/dL, 64.89 ± 13.11 mL/dL, and 66.03 ± 11.78 mL/dL, respectively. The neuron-specific enolase (NSE) and central-nerve-specific protein (S100*β*) levels at T5 were 53.85 ± 12.31 ng/mL and 7.49 ± 3.16 ng/mL, respectively. In terms of the number of postoperative complications, the patients in the experimental group were better than the control group under sevoflurane anesthesia, and the differences were substantial (*P* < 0.05). In conclusion, MRI images based on deep learning super-resolution algorithm have great clinical value in evaluating the degree of brain injury in patients anesthetized with propofol and the protective effect of propofol on brain nerves.

## 1. Introduction

Stroke is an acute cerebrovascular disease, including ischemic stroke and hemorrhagic stroke, in which blood cannot flow into the brain due to sudden rupture or blockage of blood vessels in the brain, eventually leading to damage of brain tissue [1]. According to statistics, cerebral hemorrhage patients in Asian countries account for 25%–55% of stroke patients. The mortality rate in the acute period is as high as 30%, and the mortality rate within one month is as high as 30%–50%. Even among those who survive, a large number of people are left with disabilities. It is a kind of disease with the highest fatality rate among acute cerebrovascular diseases and also one of the main diseases of death and disability of Chinese residents [2, 3]. At present, surgical surgery is mainly utilized for treatment. The craniotomy evacuation of hematoma is the most commonly performed operation in neurosurgery, and doctors can clear hematoma under direct vision and handle various emergencies in the operation well [4], which is widely utilized in clinical practice. It is the main surgical method for acute epidural hematoma, acute subdural hematoma, acute intracerebral hematoma, and hypertensive intracerebral hemorrhage [5]. There are often central nervous system-related complications associated with this type of surgery. If the right anesthesia drugs and anesthesia methods are selected, the brain tissue can be effectively protected after surgery, and the probability of cerebral insufficiency can be reduced. Propofol is the most commonly used intravenous sedative anesthetic in the clinic. It has the advantages of fast onset, short action time, and rapid recovery. When the brain is damaged, the central nervous system will release many inflammatory factors such as interleukin, which will cause some secondary inflammation or nerve cluster damage [6, 7]. Many studies have confirmed that propofol, as a widely used intravenous anesthetic, can inhibit the production of inflammatory cells and cerebral oxygen metabolism and has antilipid peroxidation effect, which can effectively reduce S100 *β* Protein, and other brain injury markers have a certain brain-protective effect [8, 9].

Magnetic resonance imaging (MRI) is an imaging diagnosis applied to all systems of the whole body, and the concept of diffusion tensor imaging (DTI) came out in the 1990s. As a development and optimization of diffusion-weighted imaging (DWI), it is a special form of MRI, capable of displaying and analyzing the conduction bundles of white matter in the brain without trauma [10]. DTI has abandoned the traditional animal model or autopsy research and can observe the integrity and coherence of tissue structure in vivo, which has a high adoption value in the investigation and determination of the damage degree and area of the white matter conduction bundle caused by diseases. It also has great advantages in displaying the transmission direction of nerve fiber bundles in white matter and accurate imaging of the central nervous system [11, 12]. However, due to the limitations of imaging equipment and other factors, DTI images may have problems such as slow imaging speed, low resolution, and redundant interference noise [13], which makes it impossible to accurately read the image information, posing great trouble for clinical work. In recent years, neural network algorithm based on deep learning has an excellent performance in the field of medical image super-resolution and has great potential in the research of clinical imaging. Deep learning is to extract prior knowledge from a large number of medical image data and generate high-resolution medical images from low-resolution medical images according to a large number of prior information. Super-resolution algorithm based on deep learning has broad prospects in many fields in recent years. It first evaluates the low-resolution images in general, learns and grasps the characteristic data information in the image [14], then transmits it to the high-resolution images, and minimizes the asymmetry between them and the original image. Compared with previous algorithms, the super-resolution algorithm based on deep learning can more deeply learn the association between high- and low-resolution images [15] and clearly display images to improve the accuracy of imaging.

In this study, a super-resolution image reconstruction model was built based on deep learning. This method was applied to 100 patients who received craniotomy evacuation of the hematoma. All patients were randomly divided into sevoflurane anesthesia control group and propofol anesthesia experimental group. The intraoperative and postoperative indexes of the two groups were compared, so as to prove the evaluation value of DTI images optimized by the super-resolution algorithm under deep learning on the brain protection of patients undergoing craniotomy hematoma removal with propofol.

## 2. Materials and Methods

### 2.1. Subjects

A total of 100 patients with cerebral hemorrhage hospitalized from May 2017 to May 2020 were selected, with an age range of 40–75 years, which were arranged to undergo craniotomy evacuation of the hematoma. They were randomly divided into the control group (*n* = 50) under sevoflurane anesthesia and the experimental group (*n* = 50) under propofol anesthesia. Inclusion criteria are as follows: (i) patients with the stroke-like disease diagnosed with acute cerebral hemorrhage by MRI; (ii) patients who underwent surgery within 48 hours of admission; (iii) patients classified as grade I and II by the *American Society of Anesthesiologists* (ASA); (iv) Patients with few or no complications and no serious diseases of vital organs; (v) patients with stable preoperative blood pressure and blood glucose control; (vi) patients with no hemoglobin, hematocrit, or abnormal coagulation function; (vii) patients with no history of long-term use of antidepressants and analgesics; and (viii) patients with complete clinical and imaging data. Exclusion criteria are as follows: (i) patients with abnormal liver, kidney, and lung function; (ii) patients with severe coronary heart disease and endocrine diseases; (iii) patients with mental illness or drug abuse; (iv) patients with other vascular diseases; and (v) patients lacked compliance and did not cooperate with the examination. This experiment had been approved by the Ethics Committee of the hospital. All relevant matters of the experiment had been informed to the patients themselves and their families, and informed consent had been signed.

### 2.2. Main Instruments, Materials, and Medicines

Anesthesia machine was purchased from Nanjing Vedeng Medical Co. Ltd. Monitor was purchased from Jinan Laibao (Biobase) Medical Equipment Co. Ltd. Blood gas analyzer was purchased from Jinan Qiansi Biotechnology Co. Ltd. Central nervous system-specific protein (S100*β*) kit was purchased from Shanghai Kameishu Biotechnology Co. Ltd. Neuron-specific enolase (NSE) kit was purchased from Shanghai Renjie Biotechnology Co. Ltd. Enzyme-linked immunoassay instrument was purchased from Shandong Jingdao Optoelectric Technology Co. Ltd. Centrifuge was purchased from Beijing Haiyi Technology Co. Ltd. The −80°C refrigerator was purchased from Henan Xinuo Instrument Equipment Co. Ltd. Sufentanil citrate injection (1 mL: 50 ug) was purchased from Yichang Humanwell Pharmaceutical Co. Ltd. Cisatracurium besylate for injection (5 mL: 10 mg) was purchased from Jiangsu Hengrui Pharmaceutical Co. Ltd. Remifentanil hydrochloride for injection (1 mg) was purchased from Jiangsu Enhua Pharmaceutical Co. Ltd. Propofol injection (50 mL: 500 mg) was purchased from Sichuan Guorui Pharmaceutical Co. Ltd. Sevoflurane for inhalation (120 mL) was purchased from Shanghai Hengrui Pharmaceutical Co. Ltd.

### 2.3. Craniotomy Evacuation of Hematoma

All patients fasted for eight hours before surgery and abstained from drinking for four hours before surgery. After patients entered the room, arterial heart rate, mean arterial pressure (MAP), pulse oxygen saturation (SpO_2_), and bispectral index (BIS) were routinely monitored and recorded. Intravenous injection of sufentanil (0.5 *μ*g/kg) and cisatracurium (0.2 mg/kg) was utilized for anesthesia induction. Endotracheal intubation was performed after the skeletal muscle relaxation was complete, followed by retrograde puncture and catheterization in the middle of the internal jugular vein guided by B-ultrasound, with a depth of about 15 cm. 2 mL venous blood from the internal jugular vein bulb was collected for blood gas analysis, and then mechanical ventilation was performed by connecting the anesthesia machine. Anesthesia maintenance for the control group was that patients inhaled 4%–6% sevoflurane, and that for the experimental group was propofol 4-6 mg·kg^−1^·h^−1^. Intraoperative composite remifentanil intravenous drip 0.1–0.3 *μ*g·kg^−1^·min^−1^ was given to both groups. The concentration/drop rate of sevoflurane/propofol in the control and the experimental groups were adjusted appropriately. During the operation, the surgical approach was determined according to the site of bleeding. After the skull was opened and the lateral fissure was separated under a microscope, the hematoma in the basal ganglia region was carefully removed through the insular approach, or the cortical fistula nearest to the cortex of the hematoma was selected (avoiding important functional areas as far as possible), entering the hematoma cavity. The hematoma was removed under the microscope; hemostatic materials were put into the operative cavity after strict hemostasis; and the skull was closed layer by layer. Sevoflurane inhalation/propofol infusion was discontinued after surgery, and the patient was admitted to an anesthetic care unit. When the patient's autonomic consciousness, swallowing reflex, muscle strength, and others gradually recovered, the endotracheal tube can be removed after the standard for extubation was reached.

### 2.4. Brain Metabolic Indexes

Radial artery blood (1 mL) and jugular venous blood (1 mL) were collected from the control and the experimental groups 15 minutes after endotracheal intubation (T1), 30 minutes after the operation (T2), when the dura mater was opened (T3), when it entered the insular lobe (T4), and when the operation was finished (T5). The hemoglobin (Hb), oxygen saturation (SaO_2_), arterial oxygen partial pressure (PaO_2_), and oxygen saturation in jugular venous (SjvO_2_) were recorded at each time point, so did jugular venous blood oxygen partial pressure (PjvO_2_), arterial blood glucose (Glua), lactic acid value (Laca), and jugular venous lactic acid value (Lacjv). According to Fick's law, the arterial oxygen (CaO_2_), cervical venous oxygen (CjvO_2_), cerebral oxygen uptake rate (CO_2_ER), cervical venous oxygen difference (Da-jvO_2_), cerebral glucose uptake rate (GluER), lactic acid production rate (LacPR), and lactic acid oxygen index (LOI) were calculated.

### 2.5. Marker Detection

Cervical venous blood of 5 mL from each patient was taken and placed in an anticoagulant tube, incubated for 30 minutes at room temperature, and centrifuged for 10 minutes at a frequency of 3,000 rpm, and the supernatant was discarded and stored in a refrigerator at −80°C. Enzyme-linked immunosorbent assay (ELISA) kit was left standing at room temperature for 30 minutes, and distilled water was utilized at a ratio of 1:100 to prepare washing liquid. With the backup of a new enzyme plate, 100 *μ*L of NSE and S100*β* protein standard materials were added into the well at a time. 100 *μ*L of the sample to be tested was added to the other wells, and 100 *μ*L of phosphate buffer salt solution was added to the blank group. Fifty microliter of dilution of enzyme marker was injected into all wells (except blank group). The sealing plate was fixed and stood at 37°C for 1 hour; then, the plate was washed 3–5 times. After drying, 50 *μ*L chromo graphic agent A was first injected into each hole; then, 50 *μ*L chromo graphic agent B was injected successively and placed in a dark place for 30 minutes. Finally, 50 *μ*L terminating solution was added to each hole. After the reaction was terminated, the plate was placed into the ELISA detector, and the absorbance value was measured at 450 nm wavelength.

### 2.6. DTI Examination

All patients were scanned using an Aera XQ 1.5T MRI machine (Siemens, Germany) and a 16-channel dedicated coil. Dispersion gradients were in 15 directions; single excitation spin echo-echo plane imaging was performed; and the scanning area was from the foramen magnum to the top of the skull. Scanning parameters were TR of 5381 ms, TE of 85 ms, FOV of 224 mm × 224 mm, matrix of 125 × 125, layer thickness of 3 mm, layer spacing of 1 mm, and 20 layers.

### 2.7. Deep Learning Algorithm Construction

In recent years, image super-resolution technology based on deep learning has achieved remarkable results. Nowadays, there are various super-resolution network models based on deep learning. These networks make supervised training for low-resolution images and original high-resolution images at the same time. In essence, they are a combination of different model frameworks and upsampling methods. The high resolution (HR) image reconstructed from a low-resolution (LR) image is closest to the real original image.

Usually, the peak signal-to-noise ratio (PSNR), a full reference image quality evaluation index, is utilized to measure the image quality after super-resolution reconstruction. When an image is recoded, the color value contained in the pixel changes, and PSNR is utilized to assess the amount of change in the color value. The larger the PSNR between one image and another image, the higher the approximation between the two images.

It is assumed that there is a high-quality image *S* with a size of *a* × *b* and another image *F* with interference noise; the mean-square-error (MSE) expression between the two is as follows:

(1) $$\begin{array}{c} \frac{1}{ab}={\sum_{l=0}^{a-1}\sum_{g=0}^{b-1}\left[S\left(l,g\right)-F\left(l,g\right)\right]}^{2}, \end{array}$$

where *b* represents the number of digits of the image pixel value, *a* represents the image sample size of each training batch, and *l* and *g* are a separate and physical storage structure for sorting the values of *a* and *b*, respectively. The mean square error is a measure that reflects the degree of difference between the estimator and the estimator. From the mean square error, the PSNR for the grayscale graph is expressed as follows:

(2) $$\begin{array}{c} \text{PSNR}=10*\;\log_{10}\frac{{\text{MAX}}_{S}^{2}}{\text{MSE}}, \end{array}$$

where MAX_*S*_=2^*B*^ − 1 represents the maximum number of pixels of the image. The smaller the MSE, the greater the PSNR, that is, the better the image reconstruction effect.

Structural similarity (SSIM) is an index for evaluating the similarity of two images. Its calculation method is expressed as follows:

(3) $$\begin{array}{c} \text{SSIM}\left(S,F\right)=r\left(S,F\right)\cdot h\left(S,F\right)\cdot e\left(S,F\right),\\ r\left(S,F\right)=\frac{2\lambda_{S}\lambda_{F}+H_{1}}{\lambda_{S}^{2}+\lambda_{F}^{2}+H_{1}},\\ h\left(S,F\right)=\frac{2\phi_{S}\phi_{F}+H_{2}}{\phi_{S}^{2}+\phi_{F}^{2}+H_{2}},\\ e\left(S,F\right)=\frac{2\phi_{SF}+H_{3}}{\phi_{S}\phi_{F}+H_{3}}. \end{array}$$

where *λ*_*S*_ and *λ*_*F*_ represent the mean value of *S* and *F*, respectively. *ϕ*_*S*_^2^ and *ϕ*_*F*_^2^ represent the variance of *S* and *F*, respectively. *ϕ*_*SF*_ represents the covariance of *S* and *F*, and *H*_*1*_, *H*_*2*_, and *H*_*3*_ represent fixed values. *r* is the range of pixel value, which is 0/225. *λ*_*S*_, *ϕ*_*S*_^2^, and *ϕ*_*SF*_ are calculated as follows:

(4) $$\begin{array}{c} \lambda_{S}=\frac{1}{K\times Q}\sum_{l=1}^{K}\sum_{g=1}^{Q}S\left(l,g\right),\\ \phi_{S}{}^{2}=\frac{1}{K\times Q-1}{\sum_{l=1}^{K}\sum_{g=1}^{Q}\left(S\left(l,g\right)-\lambda_{S}\right)}^{2},\\ \phi_{SF}=\frac{1}{K\times Q-1}{\sum_{l=1}^{K}\sum_{g=1}^{Q}\left(S\left(l,g\right)-\lambda_{S}\right)}^{2}. \end{array}$$

The operation process of multiscale residual network is as follows. First, low-resolution images are convolved with a 5 × 5 convolution kernel. Then, the feature images and the results released by each residual block are merged through the multiscale residual network by convolving the residual blocks. Finally, the convolutional bottleneck layer is utilized to integrate all the features in the image. Multiscale residual network is utilized to restore the resolution of the feature map of the subpixel layer to the resolution of the original image. Low-resolution images are reconstructed into high-resolution images. The operation is expressed as follows:

(5) $$\begin{array}{c} V_{1}=\vartheta \left(t_{5\times 5}^{1}*C_{n-1}+d^{1}\right),\\ U_{1}=\vartheta \left(t_{3\times 3}^{1}*C_{n-1}+d^{1}\right),\\ V_{2}=\vartheta \left(t_{5\times 5}^{2}*\left[V_{1},U_{1}\right]+d^{2}\right),\\ U_{2}=\vartheta \left(t_{3\times 3}^{2}*\left[U_{1},V_{1}\right]+d^{2}\right),\\ V^{\prime }=t_{1\times 1}^{3}*\left[V_{2},U_{2}\right]+d^{3}. \end{array}$$

where *t* and *b* represent the weight and apparent error of the function, respectively; the superscript on the right represents the layer it is in; and the subscript on the right represents the size of the convolution kernel applied in this layer. *ϑ*(*x*)=max(0, *x*) is a linear rectifying function, and [*V*_1_, *U*_1_], [*U*_1_, *V*_1_], and [*V*_2_,  *U*_2_] are series mode. *C* represents the number of feature graphs passed to the multisize residuals, that is, the first layer contains *C* feature images and the second layer contains 2*C* feature images. All feature graphs are input to the 1 × 1 layer, and their number is reduced to *C* by convolution, which means that the number of feature graphs of input and output of the multisize residual network is consistent. The multisize convolution block is utilized to obtain the features of images in different sizes. Enhancing its transmission in the multiscale residual network can obtain ideal PSNR and SSIM.

### 2.8. Image Processing

The postprocessing software of the Siemens MRI system was utilized to process the obtained data and obtain partial anisotropic images (FA). The region of interest was set as the anterior part of the posterior limb of the internal capsule and the ventral corticospinal tract of the midbrain. The FA values of the region of interest in the symmetric corticospinal tract on the axial radiographs were measured by three experienced professional radiologists, and the mean values were taken.

### 2.9. Statistical Analysis

All data in this experiment were compared between groups using SPSS 24.0. Counting data were expressed as mean plus or minus standard deviation ($\bar{\text{x}}$ ± *s*); measurement data were tested by *χ*^2^ test; and *P* < 0.05 indicated a statistically considerable difference.

## 3. Results

### 3.1. Comparison of Two Groups of General Data

In Table 1, the control and the experimental groups had no considerable differences in age, gender, weight, ASA rating, history of hypertension, blood loss, Glasgow coma scale (GCS) score, surgical site, and so on (*P* > 0.05).

### 3.2. Comparison of Hemodynamic Indexes and BIS Values at Each Time Point between the Two Groups

**Figure 1:**
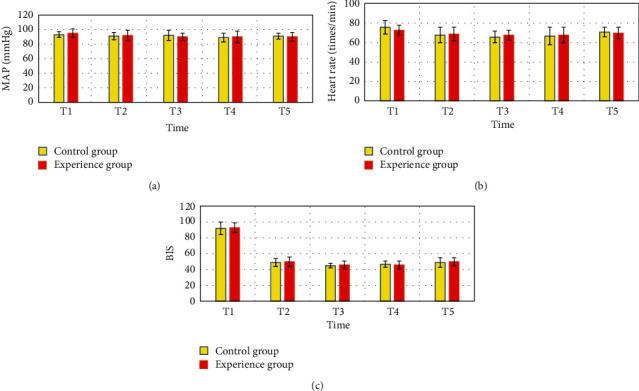
Comparison of hemodynamic indexes and BIS values of the two groups of patients at each time point: (a) the comparison of the MAP of the two groups of patients, (b) the comparison of the heart rates of the two groups of patients, and (c) the comparison of the BIS values of the two groups of patients.

In Figure 1, at each time point, there was no considerable difference in MAP, heart rate, BIS, and so on between the control and the experimental groups (*P* > 0.05).

### 3.3. Comparison of FA in the Posterior Limbs of the Internal Capsule and the Corticospinal Tract of the Ventral Midbrain between the Two Groups

**Figure 2:**
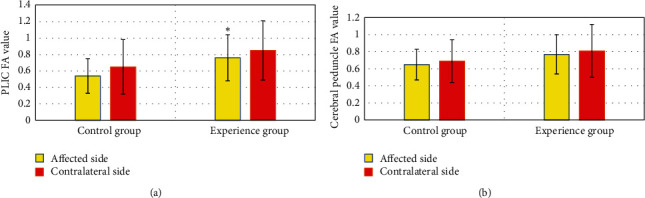
Comparison of FA values in the posterior limbs of the internal capsule and the corticospinal tract in the ventral midbrain between the two groups: (a) the comparison of FA values in the corticospinal tract of the posterior limbs of the internal capsule between the two groups and (b) the comparison of the FA values in the corticospinal tract of the ventral midbrain between the two groups of patients. ^*∗*^The FA value of the corticospinal tract of the hind limb of the internal capsule of the experimental group was remarkably different from that of the control group (*P* < 0.05).

In Figure 2, the FA value of the hind limb corticospinal tract of the internal capsule of the control group was 0.54 ± 0.21 after surgery, and that of the contralateral side was 0.65 ± 0.33. In the experimental group, the FA value of the corticospinal tract of the hind limb of the internal capsule of the affected side was 0.67 ± 0.28, and the value of the healthy side was 0.85 ± 0.36. Notably, the FA value of the experimental group was remarkably higher than that of the control group (*P* < 0.05). In the control group, the FA value of the corticospinal tract in the ventral midbrain of the affected side was 0.65 ± 0.18, and that of the healthy side was 0.69 ± 0.25. The FA value of the corticospinal tract on the ventral midbrain in the experimental group was 0.77 ± 0.23, and the value of the healthy side was 0.81 ± 0.31. There was no considerable difference (*P* > 0.05).

### 3.4. Comparison of NIHSS Scores between the Two Groups of Patients before and after Surgery

**Figure 3:**
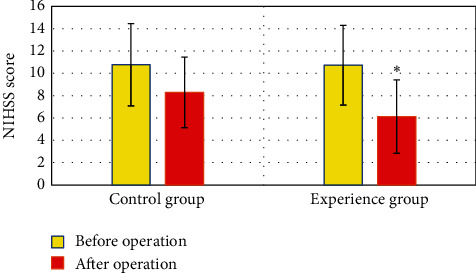
Comparison of NIHSS scores between the two groups of patients before and after surgery. Note: the black dots in the figure indicated the control group, and the red dots indicated the experimental group. ^*∗*^The postoperative NIHSS score of the experimental group was considerably different compared with the control group (*P* < 0.05).

Figure 3 shows that there was no considerable difference in preoperative NIHSS scores between the two groups of patients (*P* > 0.05). After the operation, the NHISS score of the control group was 8.31 ± 3.16 points, and that of the experimental group was 6.14 ± 3.29 points. The score of the experimental group was remarkably higher than that of the control group, and the difference was substantial (*P* < 0.05).

### 3.5. Comparison of Brain Metabolism Indexes between the Two Groups of Patients at Each Time Point

**Figure 4:**
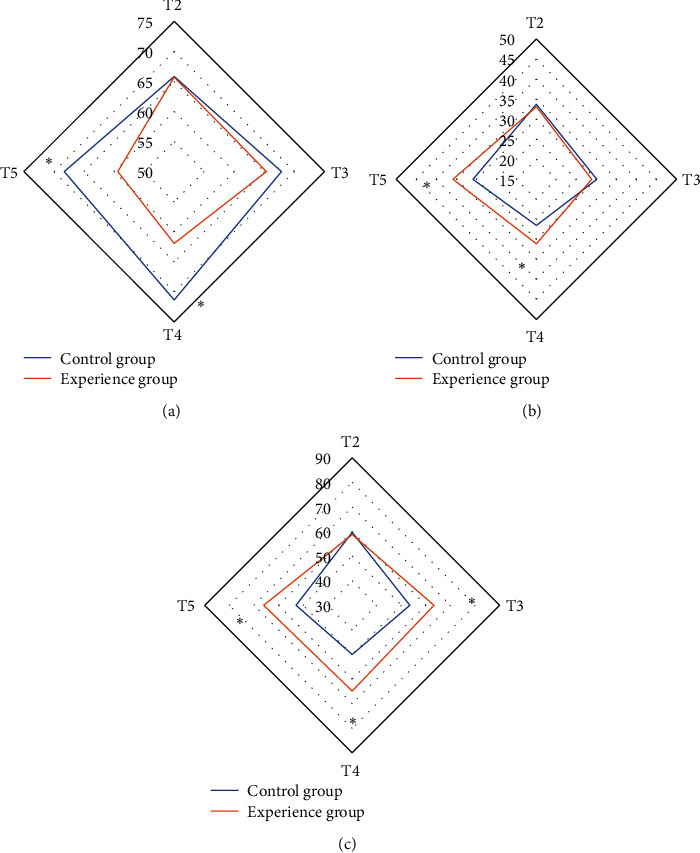
Comparison of brain metabolism indexes between the two groups of patients at each time point: (a) the comparison of SjvO_2_ at each time point of the two groups of patients, (b) the comparison of CO_2_ER at each time point of the two groups of patients, and (c) the comparison of Da-jvO_2_ at each time point of the two groups of patients. In Figure 4(a), ^*∗*^the difference in SjvO_2_ at T4 and T5 in the control group was substantial compared with the experimental group (*P* < 0.05). In Figure 4(b), ^*∗*^the difference in CO_2_ER between the experimental group and the control group at T4 and T5 was substantial (*P* < 0.05). In Figure 4(c), ^*∗*^the difference of Da-jvO_2_ at T3, T4, and T5 in the experimental group was substantial compared with the control group (*P* < 0.05).

In Figure 4, there was no considerable difference in SjvO_2_ between the two groups of patients at T2 and T3 (*P* > 0.05). SjvO_2_ at T4 and T5 in the control group were 71.34 ± 6.5% and 68.29 ± 4.7%, respectively, while that in the experimental group were 61.93 ± 6.58% and 59.38 ± 6.2%, respectively. The SjvO_2_ at T4 and T5 of the control group were higher than that of the experimental group, and the difference was substantial (*P* < 0.05). There was no considerable difference in CO_2_ER between the two groups of patients at T2 and T3, which was not substantial (*P* > 0.05). The CO_2_ER at T4 and T5 in the control group were 26.54 ± 6.18% and 30.84 ± 8.76%, respectively, and that in the experimental group were 31.12 ± 6.07% and 35.83 ± 7.91%, respectively. The CO_2_ER at T4 and T5 of the experimental group were higher than that of the control group, and the difference was substantial (*P* < 0.05). However, there was no considerable difference in Da-jvO_2_ between the two groups of patients at T2 (*P* > 0.05). Da-jvO_2_ at T3, T4, and T5 in the control group were 53.46 ± 12.58 mL/dL, 50.02 ± 11.35 mL/dL, and 52.86 ± 12.94 mL/dL, respectively, while that in the experimental group were 63.28 ± 10.15 mL/dL, 64.89 ± 13.11 mL/dL, and 66.03 ± 11.78 mL/dL, respectively. Da-jvO_2_ at T3, T4, and T5 of the experimental group were higher than that of the control group, and the difference was substantial (*P* < 0.05). From Table 2, there was no considerable difference in Hb, SaO_2_, PaO_2_, Lacjv, GluER, LacPR, and LOI between the two groups of patients at each time point, which was not statistically substantial (*P* > 0.05).

### 3.6. Comparison of NSE and S100*β* Protein between Two Groups of Patients

**Figure 5:**
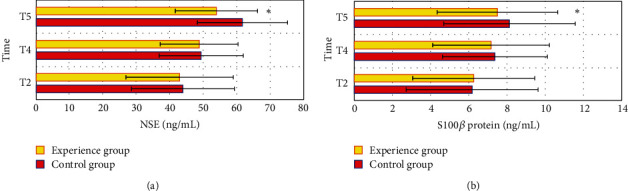
Comparison of NSE and S100*β* protein between the two groups of patients: (a) the comparison of NSE levels between the two groups of patients at each time point and (b) the comparison of the S100*β* protein levels between the two groups of patients at each time point. ^*∗*^The NSE/S100*β* protein level of the experimental group at T5 was substantial compared with the control group (*P* < 0.05).

Figure 5 shows that there was no considerable difference in NSE and S100*β* protein levels between the two groups of patients at T2 and T4 (*P* > 0.05). At T5, the NSE of the control group was 61.66 ± 13.49 ng/mL, and that of the experimental group was 53.85 ± 12.31 ng/mL. The NSE level of the experimental group was remarkably lower than that of the control group (*P* < 0.05). At T5, the S100*β* protein of the control group was 8.12 ± 3.44 ng/mL, and that of the experimental group was 7.49 ± 3.16 ng/mL. The S100*β* protein level of the experimental group was remarkably lower than that of the control group (*P* < 0.05).

### 3.7. Comparison of Various Types of Postoperative Complications between the Two Groups

**Figure 6:**
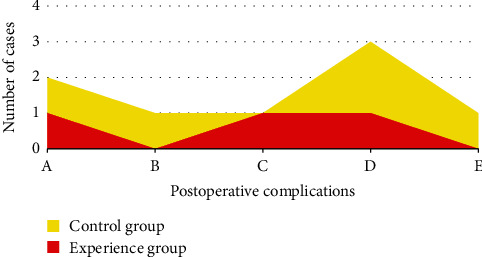
Comparison of various types of postoperative complications between the two groups of patients. Note: A meant intracranial hemorrhage, B meant intracranial infection, C meant aphasia, D meant headache, and E meant mild limb disorder. ^*∗*^The number of complications in the experimental group was considerably different compared with that in the control group (*P* < 0.05).

In Figure 6, the number of cases of common postoperative complications such as intracranial hemorrhage, intracranial infection, aphasia, headache, and mild limb disorder in the control group were 2, 1, 1, 3, and 1, respectively, while those in the experimental group were 0, 0, 0, 1, and 0, respectively. The number of complications in the experimental group was remarkably less than that in the control group (*P* < 0.05).

### 3.8. Changes in Imaging Characteristics of Patients before and after Surgery

**Figure 7:**
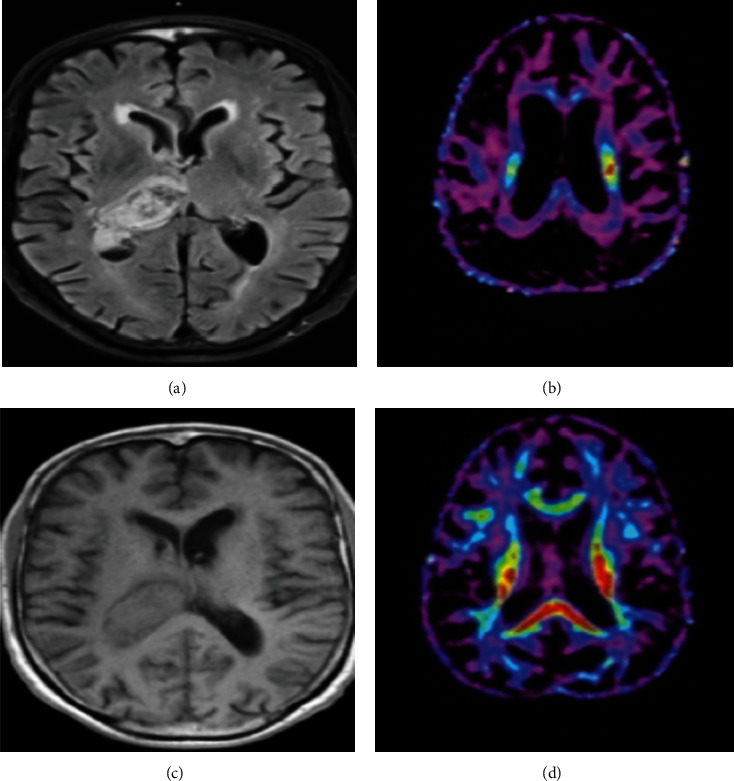
(a) Preoperative MRI images of intracerebral hemorrhage in the right brain area. The internal capsule and the anterior horn of the lateral ventricle were compressed to a certain extent. (b) DTI images of the bilateral corticospinal tracts before the operation. The conduction tracts were damaged, causing relaxation and partial disconnection. (c) One month after the operation, the hematoma was partially absorbed, and the pressure on the frontal angle of the lateral ventricle was weakened. (d) One month after the operation, the morphology of the corticospinal conduction tract was improved, and the broken conduction tract was partially recovered.

A 59-year-old male patient in the experimental group had a hemorrhage in the right brain area for two hours, with a bleeding volume of about 25 mL. The changes in MRI and DTI image characteristics before and after surgery are shown in Figure 7.

## 4. Discussion

Magnetic resonance diffusion tensor imaging (DTI) is a new technology developed on the basis of conventional magnetic resonance diffusion-weighted imaging (DWI). This technology can quantitatively measure the white matter fiber bundle of the human brain in vivo, obtain the tracer 3D imaging of the white matter fiber bundle, and more intuitively observe the damage of the white matter fiber bundle [16]. The research value of DTI in cerebral infarction, brain tumor, epilepsy, and craniocerebral trauma has been fully proved, but there is little research in craniotomy hematoma removal. Therefore, in this study, this technique was used to scan 100 patients who underwent craniotomy hematoma removal, obtain FA images, and quantitatively measure the FA values of the posterior limbs of the bilateral internal capsule and the corticospinal tract in the ventral midbrain. The degree of corticospinal tract injury can be quantitatively evaluated according to the FA values of the conduction tract in the corticospinal tract on the injured side [17]. DTI images were used to observe the conduction bundle of the affected side of the corticospinal region and intuitively evaluate the damage degree of the conduction bundle of the corticospinal region; combined with the NIHSS score before and after treatment, the comparison of brain metabolic indexes at each time point was conducted. The comparison of protein indexes and that of various types of postoperative complications were conducted; the changes of imaging characteristics of patients before and after the operation were analyzed; and the evaluation value of DTI images optimized by super-resolution algorithm was explored based on deep learning for the brain protection of patients undergoing craniotomy hematoma removal with propofol.

The common parameter FA of DTI is a parameter describing the dispersion direction of water molecules, and its value can represent the activity intensity of water molecules in the main dispersion direction [18]. The value range of FA is 0–1. In a completely uniform medium, the dispersion of water is the same in all directions, that is, the anisotropy is 0. The maximum dispersion direction of water molecules in the fiber bundle is parallel to the direction of the fiber bundle, and the anisotropy is close to 1. The corticospinal conduction tract is the largest descending fiber bundle. The main reason for limb motor dysfunction caused by hemorrhage in the basal ganglia and thalamus is to compress and destroy the corticospinal tract of the hind limb of the internal capsule. This study found that in 100 patients with craniotomy hematoma removal, the corticospinal tract in the internal capsule affected by the hematoma was reduced to varying degrees compared with the affected side. This is because the direct compression of the hematoma changed the deformation, distortion, structure, and shape of the conduction tract in the corticospinal region, and even part or all of the fiber bundles were broken, which reduced the anisotropy of the conduction tract in the corticospinal region [19].

DTI can be undertaken to observe the cerebral white matter fiber bundle in three dimensions and clearly show the shape, quantity, and damage of the fiber bundle. DTI technology has been widely used in preoperative evaluation of brain tumors, cerebral infarction, brain trauma, and so on. In this study, all patients had different degrees of damage to the conduction bundle in the corticospinal region at admission. After craniotomy hematoma removal, DTI image examination based on deep learning super-resolution algorithm showed that the number of fiber bundles in the conduction bundle in the corticospinal region on the damaged side in the experimental group increased significantly compared with that before the operation, and the recovery was better.

In this study, 100 patients with the intracerebral hemorrhage who needed craniotomy hematoma removal were divided into 50 cases of control group under sevoflurane inhalation anesthesia and 50 cases of experimental group under propofol intravenous anesthesia. DTI image examination based on deep learning super-resolution algorithm was given before and after the operation. The results showed that the FA value of the corticospinal tract area of the posterior limb of the internal capsule in the control group was lower than that in the experimental group (*P* < 0.05), indicating that the injury degree of the spinal tract area in the experimental group was less. The NHISS score of the control group was lower than that of the experimental group (*P* < 0.05). At each time node, SjvO_2_ in the control group at T4 and T5 was higher than that in the experimental group (*P* < 0.05), indicating that the degree of cerebral deoxygenation saturation in the T4 and T5 experimental groups was light and the risk of cerebral ischemia was low. Similarly, at T4 and T5, the CO_2_ER of the control group was lower than that of the experimental group (*P* < 0.05), indicating that the brain oxygen uptake rate of the experimental group was higher and the probability of brain injury was lower at this time point. The Da-jvO_2_ of T3, T4, and T5 in the control group was lower than that in the experimental group (*P* < 0.05), suggesting that the degree of secondary brain damage in the experimental group was lower at this time point. The protein level was higher than that in the experimental group (*P* < 0.05). The number of postoperative complications in the experimental group was also less than that in the control group (*P* < 0.05). MRI/DTI images of an experimental group also showed that one month after the operation, the hematoma in the affected brain area was basically absorbed, and the morphology of the fiber bundle in the corticospinal cord area recovered and improved. This is consistent with the research conclusions of Lu et al. [20], indicating that propofol as intraoperative anesthesia maintenance can effectively reduce the degree of brain injury and play a positive role in the protection of patients' brain nerves. As mentioned in the literature of Tian et al. [21], MRI images based on the deep learning algorithm have great clinical evaluation value.

## 5. Conclusion

In this study, 100 patients with cerebral hemorrhage were randomly divided into sevoflurane inhalation anesthesia group (*n* = 50) and propofol intravenous anesthesia group (*n* = 50), all of which received craniotomy evacuation of the hematoma. Before and after surgery, DTI images based on deep learning super-resolution algorithm were utilized for evaluation. The results showed that in the experimental group using propofol anesthesia, FA values, NHISS scores, brain metabolism indexes at some time points, NSE and S100*β* protein levels, and the probability of postoperative complications in the corticospinal tract of the hind limb of the internal capsule of the affected side were better than the control group anesthetized with sevoflurane, with substantial differences (*P* < 0.05). The MRI/DTI images showed that the ruptured conduction bundle in the affected side of the brain recovered quickly after surgery. However, the number of samples in this study is small, and the source is single, and the experimental results have certain limitations and one-sidedness. In the future, more samples will be collected for further investigation in this direction. It provides more reference for the evaluation value of DTI images based on the deep learning algorithm in clinical brain protection.

## Figures and Tables

**Table 1:** Comparison of general information.

	Control group	Experience group	*P*
Age	47.36 ± 10.83	46.98 ± 9.33	0.763
Male	24	25	0.732
Weight	68.72 ± 8.17	69.53 ± 7.88	0.618
ASA I	23	24	0.842
Hypertension	39	40	0.799
Blood loss	28.35 ± 7.16	27.88 ± 5.43	0.682
GCS score	9.78 ± 4.11	9.52 ± 3.89	0.788
Left-side operation	26	27	0.845

**Table 2:** Comparison of brain metabolism indexes between the two groups of patients at each time point.

Index	Group	T2	T3	T4	T5	*P*
Hb (g/dL)	Control	12.93 ± 1.21	12.87 ± 1.34	13.29 ± 1.46	13.06 ± 1.38	0.061
	Experimental	13.08 ± 1.52	12.36 ± 1.41	13.15 ± 1.5	13.11 ± 1.29	0.064
SaO_2_ (%)	Control	99.34 ± 0.41	99.12 ± 0.38	98.78 ± 0.56	99.45 ± 0.61	0.068
	Experimental	99.21 ± 0.36	99.05 ± 0.41	98.81 ± 0.42	99.35 ± 0.44	0.062
PaO_2_ (mmHg)	Control	36.89 ± 2.01	36.75 ± 1.93	37.66 ± 2.05	38.16 ± 2.17	0.063
	Experimental	37.02 ± 1.89	37.82 ± 1.97	38.94 ± 1.95	38.99 ± 2.02	0.066
GluER	Control	6.23 ± 3.01	7.08 ± 2.95	7.14 ± 3.11	6.83 ± 3.02	0.063
	Experimental	6.77 ± 2.46	7.05 ± 2.98	7.43 ± 3.05	7.58 ± 3.14	0.067
LacPR	Control	0.37 ± 0.02	0.19 ± 0.01	0.17 ± 0.02	0.22 ± 0.05	0.071
	Experimental	0.00 ± 0.01	0.05 ± 0.02	0.03 ± 0.04	0.00 ± 0.01	0.075
LOI	Control	0.04 ± 0.01	0.02 ± 0.04	0.05 ± 0.02	0.03 ± 0.01	0.058
	Experimental	0.01 ± 0.02	0.03 ± 0.02	0.02 ± 0.01	0.03 ± 0.02	0.057

## Data Availability

The data used to support the findings of this study are available from the corresponding author upon request.
